# The integrated GOR-COVID-19 health monitor: annual reporting (“long-cycle monitoring”): Christos Baliatsas

**DOI:** 10.1093/eurpub/ckac129.482

**Published:** 2022-10-25

**Authors:** C Baliatsas, A Meerdink, N Tak, M Dückers, P Geuijen, E Marra, M Bosmans

**Affiliations:** 1 Disasters and Environmental Hazards, NIVEL, Utrecht, Netherlands; 2 GGD GHOR Netherlands, Utrecht, Netherlands; 3 Impact, ARQ National Psychotrauma Centre, Diemen, Netherlands; 4 Faculty of Behavioural and Social Sciences, University of Groningen, Groningen, Netherlands; 5 RIVM, Bilthoven, Netherlands

## Abstract

**Introduction:**

Because of foreseeable COVID-19- related mental and physical health risks for the general population, a longitudinal health monitor was launched in the Netherlands. The monitoring program includes multiple methods. The current contribution places an emphasis on the so-called “long-cycle” monitoring activities at the national and regional level. The aim is to produce (bi-)annual reports on developments in health status of the general population and several potentially vulnerable subgroups (e.g. psychological and somatic comorbidities, lower socio-economic status).

**Methods:**

Primary care registrations and questionnaire data were used. Both depend on existing methods of data collection, cover a broad spectrum of health aspects and offer the possibility of pre-COVID-19-comparison. Primary care data were obtained from electronic health records of general practices in the Nivel Primary Care Database. Large-scale survey research with questionnaires was conducted in all the 25 health regions of the Netherlands among 2nd and 4th graders in high schools, adults, elderly and young adults (16-25 years old). Where possible, data were combined with data on socio-economic status and additional health aspects from Statistics Netherlands (CBS).

**Results:**

The first results from both data sources show that the COVID-19 pandemic undeniably had an effect on the mental health of youth. Compared to 2019, there was a general decrease in overall health and happiness in 2020 and a perceivable increase in social problems as well as health symptoms such as lack of smell and taste. Furthermore, a smaller groups of people experienced more psychological symptoms, serious problems like suicidal thoughts and PTSD symptomatology.

**Discussion:**

Adverse effects of the pandemic on youth are already visible in the first year after the outbreak. It is crucial to closely monitor the course of this health impact in the years to come, based on the combination of large, representative databases.

